# Economic evaluation of the practical approach to lung health and informal provider interventions for improving the detection of tuberculosis and chronic airways disease at primary care level in Malawi: study protocol for cost-effectiveness analysis

**DOI:** 10.1186/s13012-014-0195-8

**Published:** 2015-01-08

**Authors:** Elvis Gama, Jason Madan, Hastings Banda, Bertie Squire, Rachael Thomson, Ireen Namakhoma

**Affiliations:** Centre for Applied Health Research and Delivery (CAHRD), Liverpool School of Tropical Medicine, Pembroke Place, L3 5QA Liverpool, UK; Warwick Medical School, University of Warwick, Coventry, UK; Reach Trust, ᅟ, Lilongwe Malawi

## Abstract

**Background:**

Chronic airway diseases pose a big challenge to health systems in most developing countries, particularly in Sub-Saharan Africa. A diagnosis for people with chronic or persistent cough is usually delayed because of individual and health system barriers. However, delayed diagnosis and treatment facilitates further transmission, severity of disease with complications and mortality. The objective of this study is to assess the cost-effectiveness of the practical approach to lung health strategy, a patient-centred approach for diagnosis and treatment of common respiratory illnesses in primary healthcare settings, as a means of strengthening health systems to improve the quality of management of respiratory diseases.

**Methods/design:**

Economic evaluation nested in a cluster randomised controlled trial with three arms will be performed. Measures of effectiveness and costs for all arms of the study will be obtained from the cluster randomised controlled clinical trial. The main outcome measures are a combined rate of major respiratory diseases milestones and process indicators extracted from the practical approach to lung health strategy. For analysis, descriptive as well as regression techniques will be used. A cost-effectiveness analysis will be performed according to intention-to-treat principle and from a societal perspective. Cost-effectiveness ratios will be calculated using bootstrapping techniques.

**Discussion:**

We hope to demonstrate the cost-effectiveness of the practical approach to lung health and informal healthcare providers, see an improvement in patients’ quality of life, achieve a reduction in the duration and occurrence of episodes and the chronicity of respiratory diseases, and are able to report a decrease in the social cost. If the practical approach to lung health and informal healthcare provider’s interventions are cost-effective, they could be scaled up to all primary healthcare centres.

**Trial registration:**

PACTR: PACTR201411000910192

## Background

Chronic airway diseases (CAD) which include tuberculosis, asthma and chronic obstructive pulmonary disease (COPD) represent a major disease burden to most developing countries, particularly in Sub-Saharan Africa [1-4]. COPD and asthma are estimated to affect 64 million and 235 million people worldwide, respectively [3]. A diagnosis for people with chronic or persistent cough is usually delayed because of individual and health system barriers. Unfortunately, delayed diagnosis and treatment of COPD and asthma facilitates further TB transmission, severity of disease with complications and mortality. In the latest estimates, COPD was the fourth leading cause of deaths in 2004, and in 2030, it is projected to become the third leading cause, with 5.8 million or 8.6% of the total deaths [3]. It has been estimated that, asthma morbidity and mortality accounts for around 1% of all disability-adjusted life years (DALYs), equivalent to 16 million DALYs lost per year worldwide [3]. A crucial point is that all respiratory diseases, if not diagnosed, treated and managed timely and correctly, are problematic for individuals and health systems alike [5].

The WHO initiated a practical approach to lung health (PAL) strategy in early 1998 [6], a patient-centred approach to improve the quality of diagnosis, treatment and management of common respiratory illnesses in primary healthcare settings. The PAL seeks to strengthen health systems response to respiratory diseases by standardising service delivery through development and implementation of clinical guidelines and managerial support within the peripheral health system establishments. It is intended to coordinate delivery of health services among different levels of healthcare providers in the health system and between tuberculosis control and general health services [7].

Recent original studies and systematic reviews of the PAL strategy in high- and middle-income countries have demonstrated major benefits for implementing PAL on COPD [8,9]. The benefits of using the strategy include: marked improvement in TB case detection; prescription of bronchodilators and inhaled corticosteroids for asthma and COPD; reduction in complicated COPD cases. Contrary to high- and middle-income countries context, there is no clear information on the types of treatment available for both COPD and asthma that can help to control symptoms and slow disease progression in low-income countries. Similarly, current information on the cost-effectiveness of PAL is limited to randomised trial results from developed countries. Less information is available on health effects and costs of PAL interventions at population level in low-income countries [10-16]. Accordingly, the aim of this study is to provide evidence on the most cost-effective PAL interventions for COPD and asthma management in Malawi.

In most low-income countries, informal healthcare providers—a plethora of independent and largely unregulated healthcare practitioners—are a vital source of health care and often represent the first point of care for most patients [17,18]. Despite some challenges that informal providers pose to policy makers in the health sector, they also present an opportunity to address several high-priority health system concerns. It has been documented that informal providers fill gaps in formal healthcare provision and effectively reach traditionally hard to reach populations located in rural and remote areas [17,18]. This suggests that, with proper strategies, informal providers can potentially be harnessed to expand access to respiratory care through well-supported referral systems. This is in line with Van Den Boom et al. (2010) who have argued that PAL adaptation at a country level is a must and has to take into account the epidemiological, socioeconomic profile, national health policies, the structure of the health system and available health resources, especially at primary healthcare level [5].

Given that the informal health sector in Malawi is larger, a PAL strategy including informal health sector providers may lead to higher compliance to PAL intervention and thus a lower rate of COPD and their costs. This will be tested in this study. It is expected that an increased adherence to the guidelines’ recommended in PAL and PAL + informal healthcare providers will increase case finding and reduce the number of major TB, COPD/asthma complications. By reducing the number of major COPD/asthma complications, we expect a reduction in healthcare costs (e.g. hospitalisation, referral, medication), patient and family costs, costs of lost production and costs in other sectors.

### Objectives

The objective of this study is to assess the cost-effectiveness of the PAL strategy on CAD, asthma, bronchiectasis and TB, including the involvement of informal healthcare providers compared to a common strategy of treating only those with TB and not chronic airway disease. The principal research question is: Does the adoption of PAL and PAL + informal healthcare provider model of CAD management in primary healthcare settings leads to improved health outcomes and cost-effective management of patients with CAD, compared to routine primary health care?, and specific health economics research questions are:

1. What is the impact of PAL or PAL + informal healthcare providers on patient and health system costs?
2. What is the cost-effectiveness of PAL and PAL + informal provider strategies to diagnose, treat and manage CAD and TB as a routine clinical practice?
3. What is the economic feasibility of implementing the PAL or PAL + informal provider’s strategies?

## Methods and design

### Ethical approval

Ethical approval for the study was granted in Malawi by College of Medicine Research and Ethics committee (COMREC) in September 2013 and Liverpool School of Tropical Medicine Research Ethics Committee in July 2014.

### Study design

**Figure 1:**
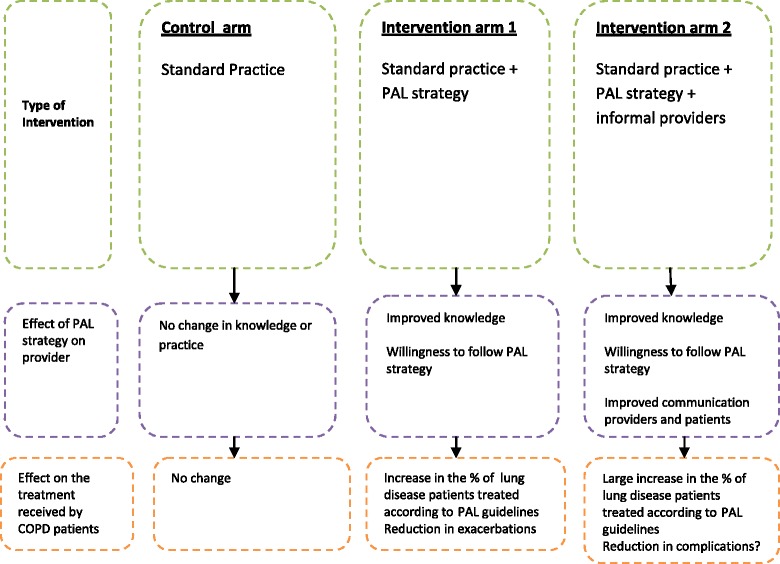
**Effect of PAL strategy on treatment received by patients.**

An economic evaluation involving three arms to compare cluster randomised controlled trial (CRCT), with randomisation at the health centre level. The randomization will be across 27 clusters, 9 clusters per arm, in two districts in Malawi. Health centre recruited to the study will be randomised to one of the three arms as indicated in Figure 1.

Health economics data collection will be conducted before and after the implementation of the intended interventions to quantify the additional costs (savings) and health gains associated with the implementation of PAL and PAL + informal healthcare providers as compared with the *status quo*. Specific secondary aims will be to determine whether the incremental costs of PAL and PAL + informal healthcare provider strategy are outweighed by incremental cost savings associated with any changes in practice (i.e. whether implementing PAL for CAD is cost saving as compared PAL + informal healthcare providers) and to determine whether PAL strategy dominates PAL + informal healthcare providers alone (i.e. less costly but of at least equivalent effect). Comparison will be made between baseline and final survey to estimate incremental cost-effectiveness ratios (ICERs) comparing the intervention with the control arms in terms of primary outcome measures (proportion of the population with a chronic cough on salbutamol/corticosteroid inhaler indicated in their health passports) and costs.

### Study population

The participants of this study will be health centres, informal healthcare providers, patients and caregivers. Interviewers will explain to all participants that involvement in the study is voluntary and they have the right to withdraw at any point in time and to ask any questions. Information about the study will be read to all participants and provided in hard copy. All consenting participants will be asked to sign two standard consent forms (that is one for the interviewee and one retained by the interviewer).

- Health centres
  Two types of health centres are included in the study: public and private-not-for-profit belonging to the Christian Health Association of Malawi (CHAM) health centres. All health centres in the eligible cluster will be enumerated and informed of the proposed study. Health centres will be selected at random and asked to provide consent prior to cluster randomisation. All health centres involved in the diagnosis and treatment of suspected cases of COPD are eligible to participate in the study.
- Informal providers
  Informal providers function within a broad and complex health system and have established some ties to other parts of the system. In particular, many informal providers have some ties with formal sector health providers. Although the nature of these interactions differs greatly by location, informal providers seem to have strong local roots and well-established long-running practices. Importantly, they are well regarded and trusted members of the community that they serve. In Malawi, for instance, more than half of the informal providers were born and live in districts where they practice. These informal providers operate as volunteers and traditional healers and offer advantages including greater flexibility, value, convenience and privacy.
- Patients and caregivers
  The inclusion of patients and their caregivers in the study is justified on the basis that these are often the only common factor moving across sites of care, and they are the most appropriate targets for an intervention designed to improve care pathways.

### Sampling

This study is planned to explore the difference in patient costs at baseline and study end, after the PAL and PAL + informal healthcare provider interventions have been implemented. At baseline, we will capture costs incurred by all patients who have CAD diagnosis in their passport. Based on the Baseline Community Survey, the expected number of patients with CAD/TB diagnosis in their passport at baseline is 135. The main effect size of the interventions is to be captured by the endline survey. The baseline survey is primarily intended to inform the sample size and design of the endline survey. This implies that power calculation and the sample size at the end of the study will be informed by estimation of patient-level variation in costs from baseline results. At a minimum, it will repeat sampling as indicated for baseline community survey.

### Trial design

Patients allocated to the control arm will receive usual clinical care, an individual intervention based on the application of the “essential health package guidelines”. These guidelines have been documented in the Ministry of Health—essential health package policy document. In addition to the standard intervention as in the control arm, patients allocated to the intervention arms 1 and 2 will be treated at facilities that have had their staff trained on PAL strategy and other in facilities that have a PAL strategy linked to informal healthcare providers in the referral system as illustrated in Figure 1.

### Outcome measures

#### Primary outcomes

1. Proportion of the population with a chronic cough who have a diagnosis of TB or airway disease(s) recorded in their health passports.

#### Secondary outcome

1. Proportion of the population with a chronic cough or salbutamol/corticosteroid inhaler indicated in their health passports.
2. Proportion of the population with a chronic cough with a diagnosis of TB or airway disease among patients with chronic cough attending primary healthcare recorded in patient registers at intervention facilities.
3. Patient costs incurred in healthcare seeking by individuals with chronic cough as well as health system resource use.

#### Additional outcome measures

The study also proposes obtaining health-related quality of life data, as measured by the Euroqol (EQ-5D-3 L) questionnaire. Data will be collected over the study period and used to generate quality-adjusted life years (QALYs) for each treatment arm. The advantage of using generic preference-based measures like the EQ-5D is that they are easy to use and can be incorporated into data collection systems easily, with little additional burden for respondents. The generic nature of EQ-5D makes them relevant to all patient groups and aids standardisation and comparisons between patient groups [19-21].

#### Costing

Economic costs will be collected from a societal perspective and will consider both the healthcare resource requirements and patient expenses associated with each treatment group. Specifically, we will collect data on three resource use: intervention costs, which include all the resources required to organise and implement CAD clinical practice; other healthcare service costs, which include the use of all health centres over the course of the trial, including both drugs and medical supplies; patient out-of-pocket expenses, which includes the individual’s own time in the treatment process and associated travel expenses. Healthcare service costs will be incurred at 18 health centres included in the intervention arms. Patient costs that will be estimated are those incurred in seeking services for CAD health care. All costs will be inflated to 2013 values, using the relevant consumer price indices from the International Monetary Fund (IMF), and converted from Malawi Kwacha to United State Dollar (US$) using relevant conversion factors from OANDA.

- Intervention costs

The project-related costs incurred between 2014 and 2015 by the two agencies implementing the project for advancing CAD-related health services in low-income settings, LSTM and REACH TRUST. The research cost associated with baseline and final surveys and research-specific technical assistance will be disaggregated from all capital and recurrent programme costs. Cost will be categorised as research or programme cost and by study arm and district and captured using an excel spreadsheet.

b) Health system costs

Primary data on provider’s costs will be collected from all health centres in the intervention arms of the study. For incremental cost analysis purposes, resource use in the control arm will be estimated basing on cost of standard care, using information from budget and financial records maintained by health centres in the control arm and their related district hospitals. In the intervention arms, data on utilisation of CAD health services, the associated consumption of drugs and medical supplies and time allocated to CAD care by staff in the health centre will be collected from individual patient records and pharmacy logs and in time allocation interviews with the health centre care providers and captured on Excel worksheet. Unit costs for healthcare resources will be derived from local and national sources and performed in line with best practices [22-27].

c) Patient costs

Data to estimate patient costs will be collected in two stages. First, through a baseline survey before the implementation of the intervention capturing costs for all patient with CAD diagnosis in their health passport. Second, a final survey 1 year after the implementation of the intervention capturing costs incurred to get treatment. The surveys will include questions on fees paid to the health system, drugs and laboratory test costs and transport, food and accommodation costs incurred as a result of the treatment process as well as time lost from economic activities due to illness or care-seeking. The STOP-TB costing tool will be adapted for use. Table 1 summarises cost data collection procedures.

**Table 1:** **Summary of data collection procedure**

**Type of cost**	**When**	**Where**	**How**	**How many**
Intervention	Ongoing-from the inception to the end of project	REACH TRUST and LSTM	Project budget and expenditure records	All data available
Healthcare service	Ongoing-from the inception to the end of project	Health centres in the intervention arms	Health centre budget and government documents	18 health centres
Patient	At baseline and at the end of project	Patient home or convenient location	Patient cost questionnaires administered at the begin and end of project	135 at baseline, sample size at endline dependent on baseline results

### Measure of resources and costs

The cost analysis will be conducted using a bottom-up approach, whereby we will determine necessary amount of each resource: personnel, equipment, material etc. and the costs per unit of each resource, then average cost will be calculated for each particular type of cost. An overview of the costs that will be measured and the corresponding sources can be found in Table 2.

**Table 2:** **Measures of health systems and patient cost**

**Measurements of cost**					
**Type of cost**	**Expenditures**	**Specification**	**Source of resources used**	**Source of cost**	**Cost calculation**
Medical direct costs	Primary healthcare consultations	Medical practitioner	Self-reported and comparison	Healthcare provider	
		Facility nurse	Answers in questionnaires		
Hospitalisation	Varied		Provider	Healthcare provider	Resource × unit cost
Secondary care consultation	Other specialist		Self-reported and comparison	Healthcare provider	Number of visits × tariff
Diagnostic tests	Radiology and laboratory		Self-reported and comparison	Healthcare provider	Number of visits × tariff
Pharmaceutical supplies	Inhalers and antibiotics		Provider	Standard pharmaceutical prices	Medicine bought × price
Additional medical services	Rehabilitation and other therapies		Provider	Healthcare provider	Service provide × tariff
Material provided in PAL intervention	Leaflets and booklets		Provider	Provider and production cost	Number of material × price
Equipment used in intervention	Sputum containers		Provider	Provider and market prices	
	Pulse oximeter				
	Nebulizer				
	Spirometer				
Non-medical direct costs	Aid to patients who face disabilities	Aid in household	Self-reported	Patient	Hours of aid × price per aid
Transport cost e.g. referrals			Self-reported	Patient	Distance × price
Indirect costs	Cost of lost	Absenteeism	Self-reported	Patient	
	Productivity				

Medical direct costs include those attributable to healthcare visits for treatment of CAD: the cost of PAL strategy utilized and training provided to healthcare providers; visits to primary healthcare facilities, to specialists and to rehabilitation services; number of essential tests; cost of medicines and disposable supplies and hospitalisation for acute episodes of asthma or COPD.

Non-medical direct costs will include home help received as a result of morbidity related to COPD, patient and guardian time directly related to the intervention (time spent getting to the facility, waiting room and intervention). Indirect costs include loss of productivity and will be calculated on the basis of time off work (absenteeism) as well as reduced productivity at work (presenteeism). The patients will be asked to quantify how much work was actually performed during regular hours and quality of this work compared to now. Cost will be calculated in international US$ following the PPP.

### Data analysis

Hypothesis being tested in the study are: 1) The practical approach to lung health strategy will decrease the incidence of CAD-related diseases and reduce the costs related to these diseases on both individuals and the health system. Thus, the PAL approach is cost-effective compared to the standard practice; 2) using both the practical approach to lung health strategy and involvement of informal providers through a referral system will be more cost-effective compared to the practical approach to lung health strategy only.

The effect of the two interventions compared with control will be analysed with methods appropriate for cluster randomised trials. To analyse the effectiveness of the PAL strategy with regard to all outcome measures (primary, secondary and QALYs), descriptive as well as regression techniques will be used. The analysis will include comparison of the three arms, as well as a multilevel analysis. The later will incorporate the levels of health worker, patient and time management. The process information regarding to what extent healthcare professional used PAL strategy will allow us to analyse the relationship between the use of PAL and the primary and secondary outcome measures. Process information will also give us insight into the experience of participants (health workers and patients), on both satisfaction and feasibility with the PAL strategy and other implementation activities.

The time horizon for inclusion of relevant costs and consequences is set as 12 months, consistent with the endline survey from the CRCT. That is to say, the economic evaluation is explicitly within trial and that any subsequent extrapolation beyond the trial period will be treated as a separate research task. Similarly, because of the short period of the study, we will not discount costs or benefits [22-26].

### Economic evaluation

For economic analysis, cost-effectiveness ratios will be calculated based on the primary outcome (i.e. the cost per case of suspected CAD that received treatment as recommended in the PAL guidelines) as well as a range of secondary outcomes including changes in provider knowledge. Cost-effectiveness will be calculated for each comparison and will be expressed as incremental cost effectiveness ratios (ICERs). In addition to ICERs, we will also calculate the cost per QALY. The economic evaluation (cost-effectiveness) will be performed according to the intention-to-treat principle and from a societal perspective. Healthcare costs (costs of diagnosis, treatment and management), costs in other sectors, patient and family costs and costs of production losses will be included. ICER will be estimated using bootstrapping techniques and graphically presented on cost-effectiveness planes. One-way and multi-way sensitivity analysis will be performed in order to assess the robustness of the results and examine the effect of varying uncertain variables on study findings. A full analysis plan will be reviewed and agreed before the data are analysed.

## Discussion

This study addresses an important problem, because CAD is among the main causes of high mortality and morbidity in communities where people lack access to diagnosis, treatment and management of the disease. This indicates that there is room for improvement of the diagnosis, treatment and management of CAD for this group of people.

PAL strategies aim to promote evidence-based practice, improve patient outcome and allow more efficient use of scarce resources. It is expected that an increased adherence to the guidelines’ recommended in PAL and PAL + informal healthcare providers will reduce the number of major COPD/asthma complications. By reducing the number of major COPD/asthma complications, we expect a reduction in healthcare costs (e.g. hospitalisation, referral, medication), patient and family costs, costs of lost production and costs in other sectors. Estimating the size of this cost reduction is an essential part of this study.

This study will also allow us to ascertain which elements of a multifaceted PAL strategy and PAL strategy + informal providers can be particularly associated with successful implementation of the PAL guidelines on CAD in low-income setting. The results of our study can contribute to more knowledge about the effectiveness of PAL strategy on CAD diagnosis, treatment and management in low-income country settings.

## Abbreviations

CAD: Chronic airways disease
COPD: Chronic obstructive pulmonary disease
CRCT: Controlled randomised clinical trial
ICER: Incremental cost-effectiveness ratio
NCD: Non-communicable disease
PAL: Practical approach to lung health
PHC: Primary health care
QALYs: Quality-adjusted life years
SSA: Sub-Saharan Africa
